# Treating insomnia during pregnancy improves bedtime procrastination, rumination, anxiety, and positive affect: a randomized controlled trial of cognitive-behavioral and mindfulness-based therapies for prenatal insomnia

**DOI:** 10.1093/sleepadvances/zpag050

**Published:** 2026-04-30

**Authors:** David A Kalmbach, Anthony N Reffi, Philip Cheng, Parisa R Kaliush, Matthew B Jennings, Heba A Afaneh, Amanda Yu, Miriam Jaziri, Christopher L Drake

**Affiliations:** Thomas Roth Sleep Disorders & Research Center, Henry Ford Health, Detroit, MI 48202, United States; Department of Obstetrics, Gynecology, and Reproductive Biology, Michigan State University, East Lansing, MI 48824, United States; Thomas Roth Sleep Disorders & Research Center, Henry Ford Health, Detroit, MI 48202, United States; Thomas Roth Sleep Disorders & Research Center, Henry Ford Health, Detroit, MI 48202, United States; Department of Psychiatry, School of Medicine, University of North Carolina at Chapel Hill, Chapel Hill, NC 27599, United States; Thomas Roth Sleep Disorders & Research Center, Henry Ford Health, Detroit, MI 48202, United States; Thomas Roth Sleep Disorders & Research Center, Henry Ford Health, Detroit, MI 48202, United States; Thomas Roth Sleep Disorders & Research Center, Henry Ford Health, Detroit, MI 48202, United States; Thomas Roth Sleep Disorders & Research Center, Henry Ford Health, Detroit, MI 48202, United States; Thomas Roth Sleep Disorders & Research Center, Henry Ford Health, Detroit, MI 48202, United States

**Keywords:** CBTI, PUMAS, sleep, rumination, meditation, cognitive arousal, maternal

## Abstract

**Study Objectives:**

Insomnia is common in pregnancy and is associated with maladaptive sleep behaviors and poor emotion regulation. Cognitive-behavioral therapy for insomnia (CBTI) and Perinatal Understanding of Mindful Awareness for Sleep (PUMAS) are effective interventions for alleviating prenatal insomnia. However, clinical benefits outside the primary target of sleep remain unclear. This randomized controlled trial (RCT) explored the effectiveness of CBTI and PUMAS on bedtime procrastination, insomnia-focused rumination, anxiety, affect, and mindfulness.

**Methods:**

Secondary analysis of a single-site, three-arm RCT of *N* = 64 pregnant women with clinical insomnia who were randomized to CBTI, PUMAS, or sleep hygiene education (SHE). Outcomes included the bedtime procrastination scale (BPS), daytime insomnia symptom response scale, generalized anxiety disorder-7, positive and negative affect schedule, expanded form-modified (PANAS positive and negative affect), and five facet mindfulness questionnaire (FFMQ). Patients provided treatment feedback.

**Results:**

CBTI and PUMAS were associated with large reductions in BPS (Cohen’s ds ≥ 1.02) and medium-to-large effects on PANAS positive affect (Cohen’s ds ≥ .60). In a subsample of patients with anxiety symptoms, CBTI and PUMAS produced large anxiolytic effects (Cohen’s ds ≥ .92). PUMAS reduced insomnia-focused rumination relative to SHE (Cohen’s d = .71), whereas CBTI did not. We observed no significant effects on negative affect or FFMQ. Patients identified behavioral sleep strategies, meditation, and honoring pregnancy as particularly helpful.

**Conclusions:**

CBTI and PUMAS yield clinical gains beyond the primary target of insomnia. These findings highlight the benefits of improving sleep quality for the betterment of overall maternal wellbeing, thereby strengthening consideration of CBTI and PUMAS as first-line treatments for prenatal insomnia.

**Clinical Trial Registration:**

Improving Negative Stressful Perseverations in Insomnia to Revitalize Expectant Moms (INSPIRE). https://clinicaltrials.gov/study/NCT04445805, National Library of Medicine ClinicalTrials.gov Registry: NCT04445805.

**Statement of Significance**

Pregnancy is a stressful period rife with significant changes in sleep patterns and behaviors. Half of women develop insomnia during pregnancy, which fuels anxiety and mood disturbances. Evidence supports Cognitive-Behavioral Therapy for Insomnia (CBTI) and Perinatal Understanding of Mindful Awareness for Sleep (PUMAS, which combines mindfulness and behavioral strategies) for alleviating prenatal insomnia. This study was a secondary analysis of a randomized controlled trial exploring whether CBTI and PUMAS yielded clinical benefits beyond sleep. Results showed that CBTI and PUMAS reduced bedtime procrastination and anxiety, and enhanced positive affect. PUMAS was associated with decreased insomnia-focused rumination, whereas CBTI was not. Trial findings build on our previously reported effectiveness results and together support CBTI and PUMAS as promising candidates for first-line prenatal insomnia treatment.

## Introduction

During pregnancy, approximately half of women experience insomnia symptoms [1–3], and one in five women meet diagnostic criteria for Diagnostic and Statistical Manual of Mental Disorders, Fifth Edition (DSM-5) insomnia disorder [4, 5]. Insomnia involves difficulty falling and/or staying asleep, leading to daytime fatigue, mood and concentration difficulties, and reduced quality of life [6]. Although sleep problems are often considered a normal feature of pregnancy, a burgeoning literature reveals prenatal insomnia to increase a wide range of negative maternal and fetal health outcomes, such as maternal depression, anxiety, and preterm birth [7–11]. To address the high prevalence and morbidity of untreated insomnia during pregnancy, recent evidence supports the effectiveness of psychotherapeutic approaches for improving sleep quality.

In recent years, empirical evidence has supported cognitive-behavioral therapy for insomnia (CBTI) for alleviating prenatal insomnia symptoms when delivered via clinicians (in-person or video) [12–15] or digital health apps [16–18]. Even so, insomnia presents differently in pregnancy, and treatment of prenatal insomnia involves navigating barriers unique to the perinatal experience (e.g. frequent awakenings due to discomfort and nocturia). As such, sleep providers and patient stakeholders have urged insomnia interventions to be tailored for the perinatal experience [19–21]. These modifications include adapting psychoeducation and behavioral sleep strategy components of CBTI to pregnancy [12, 15, 22], as well as developing pregnancy-specific interventions that combine key elements of CBTI with mindfulness to improve emotion regulation [23, 24]. An example of the latter is Perinatal Understanding of Mindful Awareness for Sleep (PUMAS).

PUMAS is a novel insomnia intervention that combines behavioral sleep strategies and mindfulness with all components tailored to pregnancy [24]. PUMAS effectively treats insomnia during pregnancy, achieving superior insomnia remission rates relative to sleep hygiene education (SHE; 81.8% vs 13.6% insomnia remission) [15]. PUMAS remission rates are comparable if not slightly higher than insomnia remission rates seen following clinician-led CBTI in pregnancy, the latter of which ranges from 41.4% to 65.0% in recent clinical trials [12, 14, 15]. Addressing a common comorbidity with prenatal insomnia, PUMAS also yields large antidepressant effects on perinatal depression in both uncontrolled and controlled trials through reducing sleep-interfering cognitive arousal at night [15, 24, 25].

Indeed, a critical advantage of cognitive-behavioral and mindfulness-based insomnia-targeting psychotherapies regards their treatment benefits that extend beyond sleep. In the general insomnia population, these interventions improve comorbid mental health conditions (e.g. depression and anxiety) and sleep-focused worry [26–29], as well as a wide range of domains spanning daily affect, emotion processing and regulation, and overall quality of life [30–33]. Early evidence suggests that cognitive-behavioral and mindfulness-based treatments for insomnia may yield similar non-sleep related benefits during pregnancy, particularly for comorbid perinatal depression [13, 15, 16, 24, 34]. However, other vital aspects of sleep and mental wellbeing in the context of perinatal sleep treatment warrant further investigation.

Prenatal insomnia is often a result of maladaptive sleep behaviors and cognitive arousal (e.g. rumination and worry) [35]. For instance, bedtime procrastination—the volitional delay of going to bed [36]—is elevated during pregnancy, particularly among poor sleepers [37]. Moreover, pregnant women with insomnia engage in high levels of ruminating about their insomnia and its consequences (e.g. ruminating about their fatigue or perceived effects on the developing baby), which, in turn, perpetuates the insomnia [8]. Anxiety is also highly comorbid with prenatal insomnia [38], and linked to maladaptive sleep behaviors (e.g. bedtime procrastination [37]) and insomnia-focused rumination [39]. Finally, in the general population, insomnia has been linked to high levels of negative affect and low levels of positive affect [40]. Positive affect, in particular, may protect against insomnia symptoms by increasing one’s engagement with pleasurable daytime activities and restorative health practices, such as going to bed on time [41].

Taken together, poorly sleeping pregnant women struggle with a host of daily challenges that extend beyond insomnia, namely a confluence of maladaptive sleep behaviors, rumination, mood disturbances, and anxiety. As described above, cognitive-behavioral and mindfulness-based treatments for the general insomnia population improve a broad range of clinical symptoms outside the primary target of sleep. By comparison, much less is known about the potential broader benefits of prenatal insomnia therapy outside insomnia and depression, representing a critical knowledge gap in this vulnerable population. While early evidence suggests that cognitive-behavioral and mindfulness-based insomnia therapies alleviate comorbid anxiety and insomnia-focused rumination during pregnancy [13, 16, 42], this evidence largely stems from uncontrolled trials. Moreover, even less is known about how cognitive-behavioral and mindfulness-based insomnia treatments may improve maladaptive sleep behaviors and affect states during pregnancy.

The present study was a secondary analysis of a three-arm parallel randomized controlled trial (RCT) examining the effectiveness of CBTI and PUMAS relative to SHE (minimal intervention control) in a sample of 64 pregnant women with clinically significant insomnia symptoms on secondary outcomes that were not powered for in the trial. We sought to explore treatment effects of CBTI and PUMAS on a wide range of insomnia- and emotion regulation-related domains including bedtime procrastination, everyday mindfulness, insomnia-focused rumination, anxiety, and daily affect.

*Hypothesis 1:* Due to the shared behavioral sleep strategies between the active treatments, we predicted that CBTI and PUMAS would produce significant reductions in bedtime procrastination relative to SHE, and that CBTI and PUMAS would not differ on bedtime procrastination.

*Hypothesis 2:* Higher levels of mindfulness are associated with lower levels of insomnia and rumination in pregnancy [43]. PUMAS focuses on cultivating mindfulness, and an uncontrolled trial has shown PUMAS to increase mindfulness [42]. Therefore, we predicted that PUMAS would be superior to CBTI and SHE for increasing everyday mindfulness.

*Hypothesis 3:* Prior clinical trials suggest that PUMAS yields large treatment effects on cognitive arousal indices during pregnancy, whereas CBTI does not [15, 18, 20, 24, 42]. Therefore, we predicted that PUMAS would be superior to CBTI and SHE for reducing insomnia-focused rumination.

*Hypothesis 4:* Based on available data from largely uncontrolled trials, we predicted that CBTI and PUMAS would be associated with decreased symptoms of comorbid anxiety during pregnancy [13, 16, 42]. Therefore, we hypothesized that CBTI and PUMAS would be superior to SHE for improving anxiety, as well as positive and negative affect states. Comparisons between PUMAS and CBTI on these affective outcomes were exploratory.

*Exploratory:* Lastly, we described patient feedback on treatment access and satisfaction including both quantitative and qualitative data.

## Materials and methods

### Ethical consideration

Ethical approval was obtained from the Henry Ford Health Institutional Review Board. Patients provided informed consent before participation. All patients were assured of anonymity, confidentiality, and the right to withdraw at any time. This study is a secondary analysis; please refer to primary outcomes report for greater details on methodology and primary outcome results regarding insomnia, depression, and cognitive arousal [15].

### Study setting, eligibility criteria, and participants

This clinical trial was conducted at Henry Ford Health, a large health system centrally located in Detroit, MI, United States. Regarding eligibility, inclusion criteria included: Insomnia Severity Index ≥11 (suggests probable DSM-5 insomnia disorder in pregnancy [5], see Measures section below); gestational age ≤ 28 weeks at enrollment; age 18–40 years; and ability to participate in telemedicine video sessions (e.g. access to reliable internet and device allowing for video sessions, ability to attend therapy privately, available when services were provided). Exclusion criteria included: prenatal complications with high risk to mother and/or fetus (e.g. pre-eclampsia; note that some “high-risk” conditions were permitted, such as diabetes and hypertension); multiple pregnancy (e.g. twins); active suicidal intent (suicidal ideation without intent was NOT exclusionary); bipolar disorder and seizure disorder (contraindicated for sleep restriction); shift work (e.g. overnight shifts); and current substance use (e.g. tobacco, alcohol, cannabis, opioids, etc.).

**Figure 1:**
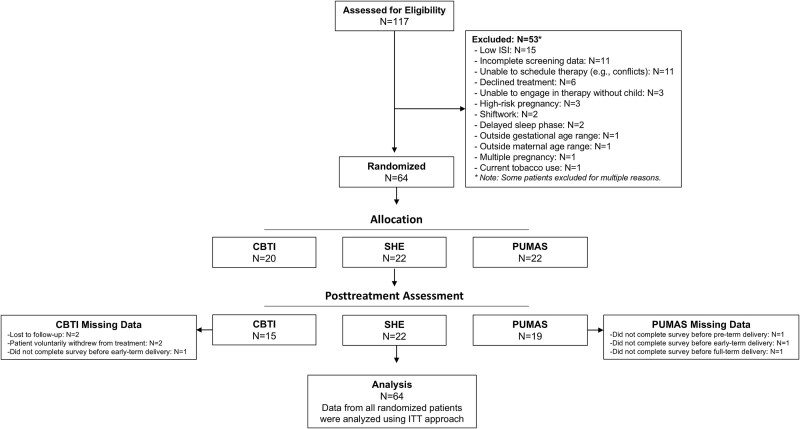
CONSORT patient flow diagram with eligibility criteria.

We emailed an advertisement for an insomnia treatment study to 3083 pregnant Henry Ford Health patients between March 2021 and July 2022. Of these invitees, 208 patients requested a call from our team to learn about the study. A total of 117 patients opened and consented to an online screening survey. Of the 108 patients who completed the screening survey, 44 patients were excluded (see Fig. 1 for Consolidated standards of reporting trials [CONSORT] Flow Diagram). The most common reason for exclusion was subclinical insomnia. Seven patients were excluded due to insufficient therapist availability (these patients were unable to be scheduled with a therapist within the gestational age limit) and another five were excluded due to scheduling conflicts (inability to participate in telemedicine appointments during times when services were provided). An additional five patients were not enrolled in the study because they were non-responsive to our contact attempts. In total, 64 women were enrolled into the RCT; see Analysis Plan below for sample size justification.

### Procedures: randomization, study outcomes assessment schedule, and intervention length

See CONSORT flow diagram in Fig. 1. Screening surveys served as baseline pretreatment values for clinical end-points, sociodemographics, and other health information. After completing the screening survey, eligible patients were contacted for enrollment into the trial. Patients were randomly assigned to PUMAS, CBTI, or SHE. We used block randomization with block size of six and a 1:1:1 allocation ratio. A project coordinator uninvolved in hypothesis generation or data analysis generated randomization allocation, enrolled patients, and assigned patients to treatment. Patients were not blinded to intervention, but they were not informed of study hypotheses or whether their treatment was considered active or control.

Patients were scheduled for their first treatment session within a week of study enrollment. Those receiving active treatment (CBTI, PUMAS) were treated in one-on-one therapy sessions. Each condition consisted of 6 weekly contacts, although patients were permitted to extend treatment beyond 6 weeks for rescheduling purposes as needed. Patients provided posttreatment assessment data 1 week after completing treatment. See Fig. 1 for CONSORT flow diagram.

### Study interventions

*CBTI* was delivered via 6 weekly 60-min telemedicine video sessions. The treatment protocol included behavioral components (sleep restriction and stimulus control), cognitive therapy, progressive muscle relaxation, sleep hygiene, and sleep education; see Table 1 for session-by-session overview. CBTI is efficacious for prenatal insomnia when delivered via clinician (in-person or telemedicine) or automated digital health app [12, 15, 16, 18, 44]. Aligning with prior CBTI trials in pregnant women, sleep education materials were tailored for the perinatal period, and we modified sleep restriction such that time-in-bed was 30 min longer than total sleep time (per Manber et al., 2019 [12]) and patients were never prescribed a time-in-bed window fewer than 6 hours (per Kalmbach et al., 2020 [18]).

**Table 1:** Brief Summary of Session-by-Session Content for Each Condition

**CBTI overview**	
***S1: Psychoeducation and sleep hygiene*** Perinatal SHE, two process model of sleep and wake, and create wind-down routine.	***S4: Cognitive therapy*** Titrate sleep schedule, review stimulus control. Discuss dysfunctional sleep beliefs, review ABCD Method and thought worksheet.
***S2: SRT*** Review 3P model of insomnia and review sleep diaries. Start SRT, discuss “enjoying your morning” handout.	***S5: Cognitive therapy (continued) and relaxation*** Review sleep strategies, review cognitive therapy homework. Assign constructive worry time and progressive muscle relaxation.
***S3: Stimulus control*** Review sleep diaries and titrate sleep schedule if necessary. Introduce stimulus control and review “things to do if you are awake” handout.	***S6: Relapse prevention*** Review homework, including constructive worry and relaxation. Review helpful components of CBTI and discuss early signs of sleep problems. Create an Insomnia Relapse Prevention Plan.
**PUMAS overview**	
***S1: Mindfulness intro & sleep hygiene*** Perinatal SHE, introduction to mindfulness, applying mindfulness to sleep, and create wind-down routine.	***S4: Mind–body & awareness of unpleasantness*** Review homework. Practice Body scan for discomfort, leading into mindfulness promotes *choosing* how we respond to unpleasantness. Assign mindfulness awareness of *unpleasant experiences worksheet*.
***S2: Step out of autopilot & SRT/sleep consolidation*** Thought metaphor & stepping out of autopilot, review meditation practice and sleep diary, start SRT/sleep consolidation.	***S5: Acceptance & letting go*** Reflect on changes in acceptance & letting go. Review unpleasant events, and explore how acceptance and letting go apply to unpleasant experiences in diary. “It is what it is” meditation.
***S3: Compassion, nocturnal thinking, pleasant events, & stimulus control*** Explore self-compassion, review homework, adjust sleep schedule, introduce stimulus control, assign mindful awareness of pleasant events worksheet, introduce nighttime meditations.	***S6: Gratitude & relapse prevention*** Gratitude meditation, review homework including progress with acceptance of unpleasant events, reflect on overall program progress and identify helpful components of PUMAS. Create action plan for insomnia (identify early signs, how to respond to insomnia).
**SHE**	
***S1: Introductory psychoeducation*** Education on why sleep matters and how much sleep is enough. The effect of poor sleep on cognition, mood, health, and safety (e.g. drowsy driving). Introduce sleep hygiene.	***S4: Diet & exercise*** Healthy eating habits, pre-sleep eating education, how diet & sleep affect health, how exercise helps sleep, and how sleep and exercise benefit overall health.
***S2: Myths about sleep, & light exposure*** Adequate sleep opportunity, snoring and signs of sleep breathing problems, psychoeducation on insomnia.	***S5: Napping, alertness boosters, & sedatives*** Effects of napping, stimulants (e.g. caffeine), and sedatives (e.g. antihistamines) on perinatal sleep and insomnia.
***S3: Healthy sleep environment*** Creating a sleep-conductive environment that is comfortable, quiet, dark, and cool.	***S6: Sleep disorders*** Psychoeducation on sleep disorders (e.g. OSA, Restless Legs Syndrome).

SRT = sleep restriction therapy. 3P model refers to precipitating, predisposing, and perpetuating behaviors of insomnia. SHE was adapted from the NHLBI’s “Your Guide to Healthy Sleep.”

*PUMAS* was delivered via 6 weekly 60-min telemedicine video sessions. Influenced by mindfulness-based therapy for insomnia (MBTI) [27, 45, 46], PUMAS combines behavioral sleep strategies with mindfulness and acceptance-based exercises. PUMAS includes elements of CBTI (sleep restriction/sleep consolidation, stimulus control) and mindfulness-based interventions (meditations, mindful activities) while tailoring these strategies to pregnancy (see Table 1 for session-by-session content overview). Please refer to our proof-of-concept trial report for greater details on how PUMAS components were tailored for pregnancy [24].

*SHE* served as a minimal intervention control. Sleep education and hygiene content aligned with the NHLBI’s *Your Guide to Healthy Sleep* [47] (see Table 1 for content). To better approximate real-world care practices where sleep hygiene and educational materials are typically distributed to patients via information sheets (e.g. in after-visit summaries), SHE was delivered digitally via information sheets. Patients randomized to SHE received 6 weekly emails, which included general, non-personalized information on the following topics: sleep regulation; the impact of sleep on health; the effects of stimulants and other sleep-disruptive substances; the relationship between sleep, diet, and exercise; and tips on creating a sleep-conducive sleep environment. Although sleep hygiene can improve sleep quality, it is not sufficient as a standalone intervention for clinical insomnia. Despite this lack of effectiveness, pregnant women report that sleep education is the most commonly delivered non-pharmacologic intervention when reporting insomnia to their providers [48].

### Measures

Study outcomes were assessed online via surveys hosted by Qualtrics. Clinical end-points of interest in this secondary analysis included bedtime procrastination, mindfulness, cognitive arousal, anxiety, and affect. These outcomes were assessed at baseline (a week before treatment) and posttreatment (a week after treatment).

*Sociodemographic information* was assessed at baseline. Patients reported age, race, household income, pregnancy health information, and medications.

*Obstructive sleep apnea (OSA)* was assessed via peripheral arterial tonometry home sleep tests (HSTs). Eligible patients were mailed a WatchPAT One device before treatment, which estimated apnea hypopnea index (AHI) per 3 per cent oxygen desaturations. OSA was operationalized as AHI ≥ 5. OSA was not used for eligibility purposes nor as a treatment outcome but rather was assessed to best describe the nature of the sample.

*Treatment engagement* was assessed via session attendance with six sessions being the maximum number of sessions across all conditions. For PUMAS and CBTI, attendance was recorded by therapists. For SHE, patients were required to open links to the weekly sleep hygiene information, which concluded with patients affirming that they reviewed the material.

*Insomnia Severity Index (ISI)* measured insomnia symptom severity [49, 50]. As previously reported, ISI was a primary clinical end-point in this RCT [15]. Scores range from 0 to 28 with higher scores reflecting greater insomnia. The ISI was used for eligibility screening with scores ≥11 suggesting the presence of DSM-5 insomnia disorder during pregnancy with .79 sensitivity and .94 specificity [5, 50].

### Measures: secondary outcomes

*Bedtime procrastination scale (BPS)* measured bedtime procrastination (e.g. “I go to bed later than I had intended”) [51]. Scores range from 9 to 45 with higher scores reflecting greater bedtime procrastination.

An 18-item version of the *five facet mindfulness questionnaire (FFMQ)* measured five facets of mindfulness: observing, describing, acting with awareness, non-judging, and non-reactivity [52]. The FFMQ can be scored as a unitary construct for overall everyday mindfulness or by each of the five facets. Higher scores on the FFMQ indicate greater mindfulness.

The *daytime insomnia symptom response scale (DISRS)* consists of 20 items assessing rumination on daytime insomnia symptoms (e.g. “Think about how tired you feel”) [53]. DISRS scores range from 20 to 80 with higher scores indicating greater insomnia-focused rumination.

The *generalized anxiety disorder-7 scale (GAD-7)* measured symptoms of generalized anxiety disorder (e.g. “Worrying too much about different things”) [54], which is a common anxiety disorder that predominantly involves excessive and persistent worry across several life domains [6]. GAD-7 scores range from 0 to 21 with higher scores indicating greater anxiety symptoms.

A 10-item shortened, modified version of the positive and negative affect schedule—expanded form (PANAS) [55] was administered to broadly assess positive affect (joyful, cheerful, happy, lively, proud) and negative affect (sad, afraid, scared, miserable, mad). On a 1–5 scale, respondents rated the extent to which they felt each affective state. In this trial, we used mean item scores for each scale with higher scores indicating greater positive or negative affect.

### Measures: patient feedback

Patient feedback included quantitative and qualitative items on the posttreatment survey. The feedback items were generated by the Principal Investigator (D.A.K.) to assess patient views on treatment delivery, treatment engagement and satisfaction, and to identify which components patients found helpful and unhelpful. Specifically, patients were asked:

Treatment access: *How difficult is it to attend routine in-person health appointments during pregnancy?* Response options included: Somewhat or much more difficult during pregnancy; Not more or less difficult during pregnancy; Somewhat or much easier during pregnancy.

Treatment delivery: *How much did the telemedicine video factor into your decision to engage in treatment?* Response options included: None to Minimal; Medium; Large.

Treatment satisfaction: *How satisfied are you with your sleep treatment during pregnancy?* Response options included: Very Dissatisfied; Dissatisfied; Satisfied; Very Satisfied.

Treatment engagement: *How engaging was your treatment during pregnancy?* Response options included: Not at all or minimally engaging to moderately or very engaging.

Improvement in nighttime cognitive arousal: *Did the sleep treatment improve your nighttime worries or nighttime stressful thoughts?* Response options included: Not at all or minimally improved; Moderately improved; Very much improved.

Helpful components: *What components or aspects of your treatment did you find most helpful during pregnancy?* Responses were open-ended.

Unhelpful components: *What components or aspects of your treatment did you find least helpful or that you think need to be improved to better improve sleep while pregnant?* Responses were open-ended.

### Analysis plan

Online surveys were hosted by Qualtrics. All analyses were performed using SPSS 26 (IBM Corp). We first examined descriptive data for sample characteristics. In addition, we utilized one-way analysis of variance (ANOVA) models and chi-square analyses to explore group differences on sociodemographics, pretreatment symptoms, and treatment adherence.

The study was powered for the primary outcome (insomnia) and recruitment was stopped when this outcome reached statistical significance in an interim analysis. Therefore, it was not powered for outcomes examined this secondary analysis report. We originally proposed a *N* = 120 sample (*n* = 40 in each condition), which was estimated to provide β > .80 power to detect a medium-large effect for independent samples group comparisons. Because this RCT was intended to collect preliminary evidence to support a funding application for a larger RCT, we planned an interim analysis after treating 50 per cent of planned sample with a Go/No-go criteria to end recruitment if significant effects were observed between active therapies and control on the primary outcome at the α = .05 significance level, or to continue recruitment if significant effect on the primary outcome was not observed. Because we observed large treatment effects of the active therapies relative to control at interim analysis, we discontinued recruitment, resulting in a final sample of *N* = 64.

Because we were specifically interested in group-to-group comparisons in this secondary analysis, we estimated power for post hoc group comparisons based on power calculations for independent samples t-tests for a two-tailed significance test at α = .05. With a minimum degrees of freedom of 40, we had β = .71 to detect large group differences (Cohen’s d = .80), β = .54 to detect medium-large group differences (Cohen’s d = .65), and β = .35 to detect medium group differences (Cohen’s d = .50). As such, to mitigate risk for committing type II errors in the post hoc group comparisons, we conducted post hoc Fisher’s least significant difference (LSD) comparisons to test significance of specific group differences, which is a less conservative test than the Bonferroni approach as this trial was not powered to detect effects on secondary clinical outcomes.

*Treatment effectiveness* was analyzed using an intent-to-treat approach using regression imputation for missing posttreatment data (see Supplementary Material). To test treatment effectiveness, we used SPSS 26 to first run 3 × 2 ANOVAs to examine Treatment × Time interactions for changes in clinical symptoms while adjusting for session attendance and relevant covariates described in the Results. After testing Treatment × Time interaction effects, we conducted cross-sectional one-way ANCOVAs to compare posttreatment symptoms by treatment condition while adjusting for covariates. For significant ANCOVA models, we conducted post hoc LSD comparisons to test group differences. We presented Cohen’s d effect sizes to describe the magnitude of significant group differences after treatment on clinical outcomes.

Treatment effectiveness for anxiety symptoms was evaluated in a subsample of patients with mild or worse anxiety symptoms before treatment. Before treatment, nearly half of patients reported “minimal anxiety” symptoms per GAD-7 classification guidelines. To address this floor effect, we considered analyzing patients who screened positive for probable clinical anxiety before treatment (GAD-7 ≥ 10). Although anxiety was highly comorbid in this sample (*n* = 9/64; 14.1 per cent comorbid anxiety), the *n* = 9 subsample size was too small for meaningful analysis. Therefore, we analyzed patients with GAD-7 scores at the sample median or higher (GAD-7 scores ≥6; *n* = 34), corresponding to mild or worse anxiety symptoms.

*Patient feedback*. Quantitative data were described using frequency rates. Qualitative data were described using direct quotes from all participants and reporting frequency rates of cited helpful and unhelpful components. In the main text, we feature quotes that were particularly representative. In the supplement, we include all quotes from all participants in the CBTI (Supplementary Table S1) and PUMAS (Supplementary Table S2) conditions.

## Results

### Patient characteristics

Sixty-four women aged 20 to 39 years participated in this RCT. Most patients were married or in a committed relationship (90.6 per cent) with 54.7 per cent of patients having previously given birth. Patients who identified as non-Hispanic white (46.9 per cent) or non-Hispanic Black (20.3 per cent) were well-represented in the sample. Over a quarter (28.1 per cent) of the sample tested positive for OSA (ranging from mild to moderate severity), and over-the-counter (OTC) sleep aid use was 7.8 per cent, whereas no patients endorsed prescription sleep aid use. Session attendance was good in all conditions (M ± SD sessions completed: 5.42 ± 1.18). In exploring relevant sociodemographic covariates, associations between lower annual income (<$50 K) with longer bedtime procrastination (*p* = .064) and lower positive affect (*p* = .068) were non-significant trends. As such, we adjusted models for annual income when predicting these outcomes in models below due to its significance level below *p* = .10. See Table 2 for summary of patient characteristics. Neither OSA diagnosis nor OTC sleep aid use was associated with baseline symptoms or posttreatment outcomes, and therefore were not adjusted for in models below.

**Table 2:** Sociodemographics, Perinatal Health, and Treatment Engagement for the Full Sample and by Group (*N* = 64)

	**Full sample**	**SHE**	**CBTI**	**PUMAS**	
Sample size	64	22	20	22	
Age	30.52 ± 4.43	30.77 ± 3.75	29.40 ± 4.72	31.41 ± 4.43	*F*(2,61) = 1.18, *p* = .314
Gestational week	26.41 ± 1.30	26.27 ± 1.42	26.55 ± 1.28	26.45 ± 1.30	*F*(2,61) = 0.24, *p* = .791
Multiparous	35; 54.7%	13; 59.1%	10; 50.0%	12; 54.5%	χ^2^ = 0.35, *p* = .840
BMI	30.10 ± 7.34	29.70 ± 7.20	30.47 ± 7.94	30.15 ± 7.24	*F*(2,61) = 0.06, *p* = .944
OSA (AHI ≥ 5)	18; 28.1%	5; 22.7%	6; 30.0%	7; 31.8%	χ^2^ = 0.50, *p* = .779
Current OTC sleep aid use	5; 7.8%	0; 0.0%	3; 15.0%	2; 9.1%	χ^2^ = 3.35, *p* = .187
Race					χ^2^ = 0.86, *p* = .650^*^
White	30; 46.9%	9; 40.9%	9; 45.0%	12; 54.5%	
Black	13; 20.3%	5; 22.7%	6; 30.0%	2; 9.1%	
Middle Eastern	5; 7.8%	2; 9.1%	1; 5.0%	2; 9.1%	
South Asian	4; 6.3%	2; 9.1%	1; 5.0%	1; 4.5%	
Eastern Asian	3; 4.7%	1; 4.5%	0; 0.0%	2; 9.1%	
Native American	3; 4.7%	1; 4.5%	2; 10.0%	0; 0.0%	
Latina	3; 4.7%	2; 9.1%	0; 0.0%	1; 4.5%	
More than one race	3; 4.7%	0; 0.0%	1; 5.0%	2; 9.1%	
Relationship status (*n*,%)					χ^2^ = 0.01, *p* = .993^†^
Married	48; 75.0%	15; 68.2%	15; 75.0%	18; 81.8%	
Unmarried, in a relationship	10; 15.6%	5; 22.7%	3; 15.0%	2; 9.1%	
Single	4; 6.3%	2; 9.1%	1; 5.0%	1; 4.5%	
Separated or divorced	2; 3.1%	0; 0.0%	1; 5.0%	1; 4.5%	
Annual household income					χ^2^ = 0.32, *p* = .993^‡^
$0 to 20 000	5; 7.8%	2; 9.1%	1; 5.0%	2; 9.1%	
$20 000 to 50 000	13; 20.3%	4; 18.2%	7; 35.0%	2; 9.1%	
$50 001 to 75 000	9; 14.1%	3; 13.6%	4; 20.0%	2; 9.1%	
$75 001 to 100 000	13; 20.3%	4; 18.2%	2; 10.0%	7; 31.8%	
$100 000 or more	24; 37.5%	9; 40.9%	6; 30.0%	40.9%	
Employment status					
Full-time	39; 60.9%	14; 63.6%	9; 45.0%	16; 72.7%	
Homemaker/Stay at home parent	11; 17.2%	3; 13.6%	6; 30.0%	2; 9.1%	
Part-time	9; 14.1%	4; 18.2%	2; 10.0%	3; 13.6%	
Student	2; 3.1%	0; 0.0%	1; 5.0%	0; 0.0%	
Temporary medical leave	2; 3.1%	1; 4.5%	0; 0.0%	1; 4.5%	
Unemployed, looking for work	1; 1.6%	0; 0.0%	1; 5.0%	0; 0.0%	
Sessions completed	5.42 ± 1.18	5.09 ± 1.60	5.30 ± 1.13	5.82 ± 0.50	H = 3.02, *p* = .213

Gestational week was assessed a pretreatment eligibility screening. BMI = body mass index at time of screening. AHI = apnea hypopnea derived from home sleep test before treatment. OSA was operationalized as AHI of 5 or higher. *p* = significance value. *F* = *F*-ratio omnibus test for ANOVA. H = Kruskal–Wallis H test. χ^2^ = chi-square test.

^*^Chi-square analysis comparing proportions of patients who identified as white vs minority across groups with the non-significant test indicating that minority rates did not differ across groups.

^†^Chi-square analysis comparing proportions of patients in a relationship (married or never married but in a relationship) vs those not in a relationship (single and separated or divorced) with the non-significant test indicating that relationship rates did not differ across groups.

^‡^Chi-square analysis comparing proportions of patients in poverty across groups with the non-significant test indicating that poverty rates did not differ across groups.

### Effectiveness: bedtime procrastination

A repeated measures ANCOVA evaluating changes in BPS scores from pretreatment to posttreatment (adjusting for income and session attendance) showed a significant Treatment × Time interaction (*F*[2,59] = 5.33, *p* = .007). A one-way ANCOVA model revealed significant group mean differences in BPS scores after treatment (Table 3) when controlling for pretreatment BPS (*p* < .001), session attendance (*p* = .020), and annual income <$50 K (*p* = .260). Pairwise comparisons showed that patients in PUMAS and CBTI reported significantly lower BPS scores than SHE patients after treatment (Table 3), corresponding to large effects for both CBTI (Cohen’s d = 1.02) and PUMAS (Cohen’s d = 1.08) relative to SHE. CBTI and PUMAS patients did not differ on posttreatment BPS.

**Table 3:** Results from One-Way ANOVA (Pretreatment) and ANCOVA (Posttreatment) Models Comparing Symptoms Across Treatment Conditions using an Intent-to-Treat Approach (*N* = 64, except where noted)

	Pretreatment *N* = 64	Posttreatment *N* = 64
**BPS**	*F*(2,61) = 0.91, *p* = .406	*F*(2,58) = 6.58, *p* = .002
SHE	26.18 ± 9.34	25.41 ± 8.26^*^^,^^†^
CBTI	26.55 ± 7.09	17.90 ± 6.40^‡^
PUMAS	23.41 ± 8.31	18.09 ± 4.95^‡^
**FFMQ**	*F*(2,61) = 2.17, *p* = .123	*F*(2,59) = 0.59, *p* = .556
SHE	59.86 ± 8.82	63.50 ± 9.23
CBTI	64.95 ± 8.23	66.55 ± 9.82
PUMAS	60.68 ± 8.20	66.82 ± 8.96
**DISRS**	*F*(2,61) = 0.81, *p* = .452	*F*(2,59) = 4.67, *p* = .013
SHE	43.05 ± 12.40	40.59 ± 11.20^†^
CBTI	46.50 ± 12.24	37.05 ± 11.70
PUMAS	42.18 ± 10.00	33.82 ± 7.49^‡^
**GAD-7 (*n* = 34)**	*F*(2,31) = 0.91, *p* = .414	*F*(2,29) = 4.36, *p* = .022
SHE (*n* = 13)	9.54 ± 2.88	7.23 ± 3.75^*^^,^^†^
CBTI (*n* = 10)	9.20 ± 3.19	3.60 ± 3.86^‡^
PUMAS (*n* = 11)	8.00 ± 2.57	2.91 ± 2.12^‡^
**PANAS Positive**	*F*(2,61) = 0.55, *p* = .583	*F*(2,58) = 4.97, *p* = .010
SHE	3.46 ± 0.93	3.27 ± 0.81^*^^,^^†^
CBTI	3.47 ± 0.74	3.78 ± 0.62^‡^
PUMAS	3.24 ± 0.72	3.67 ± 0.49^‡^
**PANAS Negative**	*F*(2,61) = 1.54, *p* = .222	*F*(2,59) = 2.44, *p* = .096
SHE	1.75 ± 0.54	1.74 ± 0.54
CBTI	2.11 ± 0.98	1.63 ± 0.63
PUMAS	1.82 ± 0.49	1.44 ± 0.47

PANAS Positive = positive affect measured by a modified positive and negative affect schedule. PANAS Negative = negative affect measured by a modified positive and negative affect schedule. Due to a flooring effect at baseline, GAD-7 analyses were conducted in *n* = 34 patients who reported a score of 6 or higher at baseline (i.e. median or higher). All pretreatment comparisons performed via one-way ANOVA. All posttreatment comparisons performed via ANCOVA controlling for pretreatment symptom values and session attendance; posttreatment models predicting BPS and PANAS Positive also controlled for annual income <$50 K. For significant ANCOVA models, we conducted post hoc pairwise comparisons using the LSD approach to describe specific group differences on posttreatment outcomes.

^*^Group mean significantly differs from CBTI mean at *p* < .05.

^†^Group mean significantly differs from SHE mean at *p* < .05.

^‡^Group mean significantly differs from SHE mean at *p* < .05.

### Effectiveness: mindfulness

A repeated measures ANCOVA evaluating changes in FFMQ from pretreatment to posttreatment did not show a significant Treatment × Time interaction (*F*[2,60] = 1.24, *p* = .298), thereby not revealing any significant treatment effects for mindfulness. See Table 3 showing no differences in FFMQ scores across conditions after treatment. For exploratory purposes, we ran repeated measures ANCOVA models for each of the five mindfulness facets measured by the FFMQ, which also did not reveal any significant treatment effects on observing, acting with awareness, non-judging, describing, or non-reactivity with significance values ranging from *p* = .063 (observing) to *p* = .723 (non-reactivity).

### Effectiveness: insomnia-focused rumination

A repeated measures ANCOVA evaluating changes in DISRS scores from pretreatment to posttreatment (adjusting for session attendance) showed a significant Treatment × Time interaction (*F*[2,60] = 4.06, *p* = .022). A one-way ANCOVA model revealed significant group mean differences in DISRS scores after treatment (Table 3) when controlling for pretreatment DISRS (*p* < .001) and session attendance (*p* = .075). Post hoc pairwise comparisons showed that PUMAS patients reported significantly lower DISRS scores than SHE patients after treatment (Table 3), which corresponded to a medium-large effect for PUMAS (Cohen’s d = 0.71). No other group comparisons were significant.

### Effectiveness: anxiety

In a subsample of *n* = 34 patients with minimal or worse anxiety symptoms before treatment, GAD-7 scores did not differ among groups before treatment (Table 3). A repeated measures ANCOVA testing changes in GAD-7 scores from pretreatment to posttreatment (adjusting for session attendance) revealed a Treatment × Time interaction was a non-significant trend (*F*[2,30] = 2.55, *p* = .095). Because the *n* = 34 subsample size reduced statistical power and the interaction term significance level was below *p* = .10, we continued by exploring posttreatment group differences. A one-way ANCOVA model revealed significant group mean differences on GAD-7 after treatment (Table 3) when controlling for pretreatment GAD-7 (*p* = .381) and session attendance (*p* = .635). Pairwise comparisons showed that patients in PUMAS and CBTI reported significantly lower posttreatment GAD-7 scores than SHE patients (Table 3), corresponding to large effects for PUMAS (Cohen’s d = 1.42) and CBTI (Cohen’s d = .95). PUMAS and CBTI groups did not differ on posttreatment GAD-7.

### Effectiveness: positive and negative affect

A repeated measures ANCOVA evaluating changes in PANAS positive affect ratings from pretreatment to posttreatment (adjusting for session attendance and income) showed a significant Treatment × Time interaction (*F*[2,59] = 4.56, *p* = .014). A one-way ANCOVA revealed significant group differences in posttreatment PANAS positive affect (Table 3) while controlling for pretreatment PANAS positive affect (*p* < .001), income <$50 K (*p* = .689), and session attendance (*p* = .308). Post hoc comparisons showed that PUMAS (Cohen’s d = .60) and CBTI (Cohen’s d = .71) had medium-large benefits for positive affect relative to SHE. PUMAS and CBTI did not differ on positive affect after treatment.

A repeated measures ANCOVA evaluating changes in PANAS negative affect from pretreatment to posttreatment did not show a significant Treatment × Time interaction (*F*[2,60] = 2.94, *p* = .060), thereby not revealing any treatment effect of PUMAS or CBTI on negative affect relative to SHE in this sample. See Table 3 showing no group differences in PANAS negative affect ratings after treatment.

### Patient feedback: quantitative data

Lastly, all patients who completed the posttreatment survey provided quantitative and qualitative feedback on their treatment experience (*N* = 56). Notably, 21 patients (37.5 per cent) described scheduling in-person health appointments as more difficult during pregnancy than before pregnancy. Unsurprisingly, nearly 90 per cent of the *n* = 34 CBTI and PUMAS patients described the telemedicine delivery of treatment as a medium-to-large influence on engaging in treatment (Table 4). After treatment, 33 of the 34 CBTI and PUMAS patients (97.1 per cent) endorsed that they would prefer virtual therapy over in-person therapy for sleep and mental health conditions in the future.

**Table 4:** Patient Feedback on Treatment Delivery and Experience (*N* = 56)

	*CBTI* *N* = 15	*PUMAS* *N* = 19	*SHE* *N* = 22
How much did the telemedicine video factor into your decision to engage in treatment?			
*Telemedicine treatment was a medium or large factor*	86.7%	89.5%	—
How satisfied are you with your sleep treatment during pregnancy?			
*Very Satisfied*	73.3%	84.2%	13.6%
*Satisfied*	26.7%	15.8%	59.1%
*Dissatisfied*	0.0%	0.0%	27.3%
*Very Dissatisfied*	0.0%	0.0%	0.0%
How engaging was your treatment during pregnancy?			
*Moderately or very engaging*	93.3%	100.0%	45.5%
*Not at all or minimally engaging*	6.7%	0.0%	54.5%
Did the sleep treatment improve your nighttime worries or nighttime stressful thoughts?			
*Very much improved*	60.0%	84.2%	9.1%
*Moderately improved*	33.3%	15.8%	27.3%
*Not at all or minimally improved*	6.7%	0.0%	63.6%

Patients provided feedback at the posttreatment assessment. Therefore, we only have these data from the *N* = 56 patients who completed this survey.

Regarding treatment satisfaction, 100 per cent of CBTI and PUMAS patients endorsed being “satisfied” or “very satisfied” with treatment, whereas 13 SHE patients (59.1 per cent) were “satisfied” with treatment and only three SHE patients (13.6 per cent) were “very satisfied” with their intervention (Table 4). Similarly, most CBTI and PUMAS patients rated their treatment as moderately to very engaging, whereas only 45.5 per cent of SHE patients described the treatment this way (Table 4). Regarding cognitive arousal, most PUMAS patients described their nighttime worries and stressful thoughts as very much improved (84.2 per cent), and 60.0 per cent of CBTI patients reported improved nighttime thoughts, whereas only 9.1 per cent of SHE patients reported improved nighttime thoughts. Notably, SHE was the only condition in which most patients (63.6 per cent) reported minimal to no improvement in nocturnal cognitions.

### Patient feedback: qualitative data

*CBTI:* When asked to identify helpful components, CBTI patients frequently cited prescribed sleep schedules and reduced time-in-bed (*n* = 7/15; e.g. “The sleep schedule explained to me, I’ve tried implementing something similar in the past but didn’t know how to go about it.” [*sic*]) and progressive muscle relaxation (*n* = 3/15; e.g. “I think the progressive muscle relation and keeping a schedule was the most helpful for me” [*sic*]) as helpful components of treatment.

By comparison, no unhelpful components were cited more than once in CBTI. Even so, we highlight important patient concerns regarding whether CBTI was sufficiently tailored to pregnancy (“I think the therapy focused on sleep but there was no real advice or attention put on the pregnancy part of it.” [*sic*]), duration of treatment (“I’m not sure if a full 6 weeks of sessions was needed…”), and rigidity of the sleep schedules (“Setting standard bed and awake times. I enjoy awaking and sleeping when i would like.” [*sic*]). Please see Supplementary Table S1 for all quotes from CBTI patients.

*PUMAS:* When asked to identify helpful components, meditations were the most commonly cited helpful components of PUMAS (*n* = 17/19; e.g. “adding meditations in bed also helped lull me into sleep mode”). Several patients cited prescribed sleep schedules (*n* = 10/19; e.g. “sticking to the same bed time/wake time has been extremely helpful”), the wind-down routine (*n* = 4/19; e.g. “Developing a wind down routine”), and pleasant and unpleasant experiences diaries (*n* = 3/19; e.g. “increasing awareness/ability to be present via pleasant and unpleasant diaries”) as other helpful components.

By comparison, multiple PUMAS patients cited the following components as unhelpful or in need or improvement: journaling (*n* = 3/19; e.g. “I am not sure how the journal entries did or did not affect my sleep”) and mindful awareness of unpleasant experiences (*n* = 2/19; e.g. “I usually avoid thinking about unpleasant experiences or avoid negative thoughts…”). The following components were cited for improvement by just one patient each, but represent important areas of potential improvement: rigidity of sleep schedules (“Sticking to a strict bedtime and wake up time”) and diversifying the meditations (“I enjoy white noise/gong sounds and sometimes find a talking voice distracting.”). Please see Supplementary Table S2 for all quotes from PUMAS patients.

## Discussion

This study was a secondary analysis exploring the effectiveness of telemedicine CBTI and PUMAS on maladaptive sleep behaviors, mindfulness, rumination, anxiety, and affect in a sample of 64 pregnant women with clinical insomnia. RCT results supported the effectiveness of CBTI and PUMAS for reducing bedtime procrastination and anxiety, and for increasing positive affect. PUMAS reduced insomnia-focused rumination relative to SHE, whereas CBTI did not. Contrary to hypotheses, we observed no significant treatment effects on mindfulness or negative affect. Taken together, the benefits of prenatal insomnia therapy extend beyond previously reported improvements in sleep to also include improvements in bedtime behaviors, mood and anxiety, and the tendency to ruminate on sleep problems.

### CBTI and PUMAS were associated with reductions in bedtime procrastination

Bedtime procrastination is a volitional delay of going to bed at night (i.e. not a result of external circumstances causing the delay). Meta-analytic data show that longer bedtime procrastination is associated with greater depression, anxiety, and stress, as well as lower positive affect, self-compassion, and overall emotional wellbeing [36]. A recent cross-sectional study of 422 pregnant women suggested that bedtime procrastination is elevated among those with poor sleep quality and anxiety [37].

Emerging evidence supports psychotherapeutic approaches for reducing bedtime procrastination. Clinical trials have shown that motivational interviewing and cognitive-behavioral approaches reduce BPS scores in bedtime procrastinators without insomnia [56–58]. The present RCT builds upon these studies to highlight that insomnia therapies—likely owing to the prescribed behavioral sleep strategies, such as establishing consistent wind-down routines and sleep–wake schedules—produce large reductions in bedtime procrastination, thereby characterizing bedtime procrastination as a maladaptive sleep behavior that is modifiable during pregnancy.

### PUMAS was associated with reductions in insomnia-focused rumination

High cognitive arousal—including broadly focused and perinatal specific perseverative thinking—is central to prenatal insomnia [35], and poorly sleeping moms identify cognitive arousal as a top target for insomnia treatment [21]. Unfortunately, CBTI produces limited clinical benefit on cognitive arousal during pregnancy [15, 18, 20]. However, alleviating cognitive arousal is associated with greater treatment response to insomnia therapies [25], so our team designed PUMAS to utilize meditation and other mindfulness- and acceptance-based exercises to help moms let go of intrusive thoughts—like worry and rumination—to reduce cognitive arousal.

Insomnia-focused rumination is a maladaptive response to sleep problems that perpetuates sleep problems. In an uncontrolled study, PUMAS produced large reductions in insomnia-focused rumination [42]. The present study adds the first RCT evidence demonstrating that PUMAS yields medium-large reductions in rumination on the perceived daytime consequences of insomnia. This highlights that PUMAS effectively defuses ruminative reactions to sleep difficulties that perpetuate insomnia, which is consistent with reducing cognitive arousal to improve sleep quality in pregnancy [15, 25].

Overall, emerging evidence suggests that PUMAS produces large reductions in both broadly focused and perinatal specific perseverative thinking at night [15, 24, 42]. The present study’s findings are consistent with our previous report comparing CBTI, PUMAS, and SHE [15]. Those RCT data revealed PUMAS produced large reductions in nocturnal cognitive arousal and perinatal rumination (worrying and ruminating about pregnancy- and baby-related concerns), whereas CBTI did not yield clinical benefits for these cognitive arousal outcomes. Complementing these clinical outcomes data is the patient feedback received in the present trial. Of the three conditions, PUMAS patients reported the highest ratings of improved nighttime worries and stressful thoughts, and the majority of PUMAS patients described meditations as helpful for falling asleep (Supplementary Table S2). Together, this growing evidence supports imbuing insomnia therapy with mindfulness to enhance treatment effects on the diverse manifestations of cognitive arousal, which may be important for maximizing treatment benefits for insomnia patients presenting with entrenched worry and rumination.

### CBTI and PUMAS reduce anxiety and increase positive affect

*Anxiety* is highly comorbid with insomnia during pregnancy [38, 59]. In the present trial, 34 patients (52.1 per cent) endorsed mild anxiety symptoms or worse. Both CBTI and PUMAS demonstrated large reductions in anxiety symptoms on the GAD-7. This replicates outcome data from controlled and uncontrolled trials supporting anxiolytic effects for CBTI (digital and clinician-led) and PUMAS during pregnancy [13, 16, 42].

The term “anxiety” captures a broad array of symptoms and disorders. Available trial data for prenatal insomnia has largely focused on symptoms of generalized anxiety disorder (worry is the cardinal feature) and anxiety specifically about pregnancy and/or childbirth. Yet, other forms of anxiety are especially germane to pregnancy and insomnia during this period, but have received less research attention. For instance, meta-analytic data estimate that 9.1 per cent of pregnant women have obsessive compulsive disorder (OCD) [60], and early evidence from prospective data suggest an etiological link between prenatal insomnia and OCD [59]. Despite these findings, OCD remains understudied in prenatal insomnia research. Future studies should clarify the clinical burden of comorbid OCD in the context of prenatal insomnia, and evaluate whether insomnia therapy (e.g. CBTI or PUMAS) may alleviate comorbid OCD symptoms.

*Affect states.* Both CBTI and PUMAS improved positive affect states marked by feelings of joy and happiness, whereas no treatment effects were observed for negative affect. We are not aware of any published trial data evaluating affect changes in response to prenatal insomnia therapy, but a small number of studies have evaluated the effects of insomnia therapy on affect states in the general insomnia population. Overall, clinical trials have reliably shown that insomnia therapy increases positive affect, whereas findings regarding treatment effects on negative affect have been inconsistent [33, 46, 61]. This empirical evidence suggesting robust insomnia treatment effects on positive affect aligns with the present study’s findings supporting CBTI and PUMAS for increasing positive emotional states during pregnancy. Positive affect gains in CBTI and PUMAS may promote antidepressant effects and increased resiliency in poorly sleeping moms.

Anhedonia, marked by blunted positive affect, is the cardinal feature of depression [62]. Naturalistic observation of prospective diary data show that better sleep quality at night predicts increased positive affect the next day in women [63]. The present study results add to this literature by showing that interventions improving sleep quality engender increases in happiness and joy, which may play a central role in the documented antidepressant effects of CBTI and PUMAS [12, 15, 16]. Additionally, insomnia therapy has been shown to increase psychological resilience, which protects against future insomnia and depression [64]. Pregnant women who are able to experience positive affect during stressful life events may be prognosticated to have more positive outcomes. Future research is needed to delineate whether increasing positive affect through insomnia therapy may increase resilience for expecting moms, which may embolden mental wellbeing when faced with adversity during pregnancy and postpartum.

### PUMAS therapeutic benefits may not require improving everyday mindfulness

Contrary to hypotheses, PUMAS did not increase mindfulness, as measured by the 18-item version of the FFMQ, relative to CBTI or control. These results add to a literature of mixed findings regarding whether mindfulness-based insomnia therapies increase everyday mindfulness [23, 29, 42, 65]. As mindfulness-based insomnia therapies reliably produce strong effects on sleep and mental health outcomes [28], even without reliable effects on mindfulness [23, 29, 42, 65], it is possible that mindfulness-based approaches do not require changes in general daily mindfulness to exert clinical benefits. Rather, the six-session mindfulness bolus of PUMAS may provide patients with sufficient mindfulness skills that can be effectively deployed for sleep and cognitive arousal, but may not be sufficient to yield changes on everyday mindfulness. In other words, PUMAS may more reliably provide mindfulness tools for patients to navigate their sleep problems and nighttime ruminations, but may be less likely to transform how people move through the world.

On the other hand, mindfulness is a complex construct encompassing many conceptualizations, and the 18-item version of the FFMQ may not best capture the specific changes in mindfulness needed to improve maternal sleep and mental wellbeing. Consider that PUMAS and MBTI reliably reduce cognitive arousal in their target patient populations [24, 29, 46, 61]. While the underpinnings of such change are surely myriad (e.g. some patients meditate to respond to stress more mindfully, whereas others may use meditation as distraction), it is possible or even likely that some mindfulness patients ruminate and worry less because they learn to respond to stressful events more mindfully (e.g. more adept at disengaging from worry-laden autopilot thinking). In other words, therapy-driven changes in letting go of intrusive thoughts may not be best captured by some mindfulness measures. An alternate—but not mutually exclusive—pathway involves positive affect. Mindfulness has been proposed to exert salubrious effects via enhancing positive affect, such that mindfulness promotes serenity, which triggers an upward spiral of positive emotion [66]. In summary, changes in letting go of intrusive thoughts (observed by reduction in rumination) and increases in positive emotions may reflect changes in mindfulness that may not be best captured by measures of everyday mindfulness.

### Patient feedback

Lastly, we evaluated patient thoughts on treatment access, delivery, and satisfaction. Over one-third of patients reported increased difficulty attending in-person healthcare appointments during pregnancy, thereby supporting efforts to ease care access through telemedicine delivery. An overwhelming majority of CBTI and PUMAS patients cited telemedicine delivery as a significant factor in their ability to engage in insomnia treatment, and that they would prefer telemedicine therapy in the future for sleep and mental health treatment.

While examining treatment effectiveness based on clinical outcomes data is critical, patient feedback on treatment should also be carefully considered. All CBTI and PUMAS patients who provided feedback were satisfied with their care, whereas dissatisfaction rates were greater for SHE. As SHE is the most widely delivered non-pharmacological insomnia intervention for pregnant women receiving real-world care [48], higher treatment dissatisfaction coupled with poor effectiveness data in pregnancy [15, 18] should guide clinic leadership and provider stakeholders away from SHE initiatives to more effective approaches.

When asked to describe helpful components of treatment, CBTI patients most commonly cited sleep restriction/sleep consolidation and relaxation. PUMAS patients also commonly cited sleep restriction/sleep consolidation as helpful, although meditation was the most frequently cited helpful component of PUMAS, followed by learning to be mindfully present during pleasant experiences. Similar to our previous trial [24], being mindfully present during unpleasant experiences received mixed feedback with some patients finding therapeutic value in responding to unpleasantness more mindfully, whereas other patients preferred avoiding negative emotions (see quotes in Supplementary Table S2). Infrequent but important areas of improvement for patients included wanting treatment to be tailored better to pregnancy (CBTI feedback) and increasing the flexibility in prescribed sleep–wake schedules (CBTI and PUMAS).

### Limitations

Findings from the present study should be interpreted in light of certain methodological limitations. Most notably, the study was powered for the primary outcome (insomnia) rather than the secondary outcomes examined this manuscript. Because this was an exploratory secondary analysis on additional outcomes that were not powered in the trial, we were underpowered to detect smaller effects. Indeed, post hoc power calculations estimated that we had β = .71 to detect large group differences after treatment on these secondary outcomes, suggesting that we were underpowered to detect clinically relevant medium and medium-large effects. This highlights the preliminary nature of the study, and that significant treatment effects and statistical trends of clinical importance deserve further evaluation in larger RCTs.

A second notable limitation is the nature of the SHE group. We chose SHE as the control condition given its ubiquity as real-world care in this population [48, 67], yet our administration of SHE is a methodological limitation. In a tightly controlled efficacy trial, SHE may have been matched to CBTI and PUMAS for delivery type (i.e. clinician-administered video sessions). In contrast, a trial favoring real-world implementation may have administered SHE with greater ecological validity reflecting real-world care practices (e.g. a one-shot information sheet similar to after-visit summaries). For this effectiveness trial, we attempted to find a middle ground by matching for contact frequency while presenting SHE in a real-world manner via information sheets. This compromise nevertheless opens our methodology to criticisms coming from perspectives prioritizing more methodological rigor or ecological validity.

Lastly, important limitations regarding generalizability include: Hispanic and Latina mothers were underrepresented; Lower-income patients were underrepresented; All patients were in the late second or early third trimester at study enrollment, thus, women in early pregnancy were not represented in this study. Regarding generalizability of patient feedback, we were unable to collect treatment feedback from patients who were lost to follow-up or otherwise did not complete posttreatment assessment before childbirth, which may have potentially led to positive responder bias.

### Clinical implications and future directions

CBTI and PUMAS are effective for alleviating insomnia and comorbid depression during pregnancy [12, 15, 16], thereby earning strong consideration to be first-line treatments for prenatal insomnia. Findings from this RCT suggest that patient benefits extend beyond sleep and depression such that CBTI and PUMAS were associated with improvements in comorbid anxiety, positive affect, and bedtime procrastination. Our observation that PUMAS was associated with large reductions in insomnia-focused rumination—whereas CBTI was not—is consistent with a growing literature suggesting that combining mindfulness with behavioral sleep strategies may enhance treatment effects on cognitive arousal both within pregnancy [15, 24] and in the general insomnia population [28]. Important to emphasize is that comorbid OSA did not impact treatment outcomes, thereby indicating that prenatal insomnia patients with untreated mild-to-moderately severe OSA benefit from CBTI and PUMAS on these non-sleep domains. Overall, our trial findings highlight the far-reaching benefits of improving prenatal sleep quality for the betterment of maternal wellbeing. Given the growing effectiveness data supporting cognitive-behavioral and mindfulness interventions for prenatal insomnia, pivoting toward real-world implementation is the next essential step forward. Lastly, we are not aware of any published data supporting the triaging of insomnia patients to a cognitive-behavioral vs mindfulness-based approaches. Rather, treatment recommendations should be based on provider clinical training and judgment, and patient preference.

Future studies should clarify the extent to which improving prenatal sleep confers postpartum benefits to maternal sleep and wellbeing. An emerging literature shows that CBTI during pregnancy is associated with better sleep quality and lower levels of anxiety and depression during the first six months following childbirth [68, 69]. Additional research is needed to explore whether treating insomnia with CBTI or PUMAS during pregnancy similarly benefits sleep behaviors (e.g. bedtime procrastination), emotion regulation (e.g. rumination and worry), affect states, and mother-to-infant bonding during the postpartum period. Evidence suggesting durable effects of prenatal insomnia treatment on postpartum sleep behaviors and emotion regulation may protect against insomnia, depression, and anxiety after childbirth. Similarly, mothers who developed insomnia during pregnancy may be vulnerable again in future pregnancies. Exploring whether prenatal insomnia care may reduce the risk of developing insomnia or related mental health conditions during future pregnancies would further elucidate the potential benefits of prenatal sleep care.

## Supplementary Material

INSPIRE_1_rct_secondary_outcomes_supplement_04242026_zpag050

## Data Availability

The datasets used and/or analyzed during the current study are available from the corresponding author on reasonable request.

## Editors and editorial board members

CLD and ANR hold the position of Associate Editor for *SLEEP Advances* and have not peer reviewed or made any editorial decisions for this paper.

DAK and MBJ hold the position of editorial board member for *SLEEP Advances* and have not peer reviewed or made any editorial decisions for this paper.
