# Supplementary material for: Treating insomnia during pregnancy improves bedtime procrastination, rumination, anxiety, and positive affect: a randomized controlled trial of cognitive-behavioral and mindfulness-based therapies for prenatal insomnia
Source: Sleep Adv. 2026 Apr 30;7(2):zpag050. doi: 10.1093/sleepadvances/zpag050 (PMC13274979; doi:10.1093/sleepadvances/zpag050)
Supplement: INSPIRE_1_rct_secondary_outcomes_supplement_04242026_zpag050 [file inspire_1_rct_secondary_outcomes_supplement_04242026_zpag050.pdf]

SUPPLEMENT

Treating Insomnia During Pregnancy Improves Bedtime Procrastination, Rumination, Anxiety, and Positive Affect: A Randomized Controlled Trial of Cognitive-Behavioral and Mindfulness-based Therapies for Prenatal Insomnia

David A Kalmbach,[1,2] Anthony N Reffi,[1] Philip Cheng,[1] Parisa R Kaliush,[3] Matthew B Jennings,[1] Heba A Afaneh,[1] Amanda Yu,[1] Miriam Jaziri,[1] & Christopher L Drake.[1]

[1] Thomas Roth Sleep Disorders & Research Center, Detroit, Michigan, 48202 USA.

[2] Department of Obstetrics, Gynecology, and Reproductive Biology, Michigan State University, East Lansing, Michigan, 48824 USA.

[3] Department of Psychiatry, School of Medicine, University of North Carolina at Chapel Hill, Chapel Hill, NC, 27599 USA.

Corresponding author:
David A Kalmbach PhD
Henry Ford Health
39450 W 12 Mile Rd
Novi, MI 48377 USA
dkalmba1@hfhs.org

Analysis Plan: Regression Imputation

The main findings on the primary outcomes of this trial were published in 2025 (see Kalmbach et al., 2025 in Sleep Medicine). Primary outcomes included the insomnia severity index (ISI), Edinburgh postnatal depression scale (EPDS), and the pre-sleep arousal scale's cognitive factor (PSASC), which were assessed at pretreatment, weekly across treatment, then at posttreatment. In the main findings report, for patients missing primary outcomes data at posttreatment, we carried forward their last weekly survey values. For instance, if a PUMAS patient underwent childbirth after session 5 and therefore did not complete posttreatment assessment, then we used their ISI, PSASC, and EPDS values from session 5 as their posttreatment scores.

In the present study, we are exploring secondary outcomes, which were only measured before and after treatment (i.e., no weekly assessments throughout treatment). Of the 64 patients in this trial, we collected posttreatment data from 56 patients (87.5% retention), whereas eight patients were either lost to follow-up or gave birth before completing posttreatment assessment (see CONSORT diagram). For these eight patients, we imputed posttreatment secondary outcome values based on their last observed ISI, PSASC, EPDS scores using multiple linear regression, thereby allowing us to perform an intent-to-treat (ITT) approach to analyses.

A step-by-step detail of how we imputed outcomes using multiple linear regression: Using the bedtime procrastination scale (BPS) as an example. The initial step involved regressing posttreatment BPS on posttreatment ISI, PSASC, and EPDS in a multiple linear regression model. This model was statistically significant ($F[55]=11.85$, $p<.001$) and explained 40.6% of the variance in posttreatment BPS ($R^2=.406$). For patients missing posttreatment BPS data, we applied the regression equation to impute missing secondary outcome values: BPS=8.568+0.535(ISI)+.567(PSASC)+-.058(EPDS). For a patient who reported ISI=6, PSASC=10, and EPDS=5 (all low symptoms) at session 6 of treatment, the regression equation predicted their posttreatment BPS score to be 17. By contrast, for a patient reporting ISI=15, PSASC=25, and EPDS=13 (all high symptoms) at session 6, the equation predicted their BPS score to be higher at 30. This process was applied to all secondary outcomes in this manuscript.

Patient Feedback: Qualitative Data

After treatment, patients were asked to share their thoughts on both the helpful and unhelpful (or in need of improvement) components of CBTI and PUMAS. In the main text, we described frequently cited components for each treatment and included representative quotes, some of which were shortened in length for readability. Below in this supplement, we share all of the qualitative feedback that we received for CBTI and PUMAS. In the tables below, we present all quotes from CBTI patients (Table S1) and PUMAS patients (Table S2), which are completely unaltered from how they were reported by patients. Before presenting these patient quotes, we want to clarify a couple points: In Table S1, Patient 12 identified an unhelpful component of CBTI as a cord being too short on a sleep machine. Similarly, in Table S2, Patient 16 cited that they found wearing a sleep tracker seemed irrelevant to their care. We want to clarify that no sleep devices were used in either the CBTI or PUMAS conditions. Rather, we believe these patients were likely referencing the WatchPAT One home sleep test (HST) device, which was used for diagnostic purposes only. Lastly, we want to acknowledge that 'mediation' was entered often in patient feedback, mostly in the PUMAS condition. Given the context of these comments, we considered these to be typos and interpreted them as 'meditation.'

SUPPLEMENT

Table S1. Open-ended, qualitative patient feedback on the helpful and unhelpful components of CBTI during pregnancy (all quotes remain unaltered).

	CBTI	
	**What was helpful?**	**What was unhelpful or needs to be improved?**
Patient 1	I was able to fall back asleep faster and woke up less. I think the progressive muscle relation and keeping a schedule was the most helpful for me.	I didn't find the mediation recordings to be easy to use. I didn't want to put headphones in in the middle of the night.
Patient 2	I enjoyed the therapist sharing the results of my surveys with me, so the 'numbers' part was interesting and compelling to me	I think the therapy focused on sleep but there was no real advice or attention put on the pregnancy part of it. Even the survey questions could go a bit further because for example I am in pain and breathless now but that is because I am 8 months pregnant not because I have any other underlying sleep apnea or obesity issues. Also the actual pregnancy was not talked about during the therapy sessions very much at all, I thought it would be more pregnancy focused tips and tricks/advice. I feel like I could have not been pregnant and been in the exact same study.
Patient 3	Different tactics that could be used every week to help with falling asleep	N/A
Patient 4	The sleep schedule explained to me, I've tried implementing something similar in the past but didn't know how to go about it. It's helped me quite a bit. Also the CBT worksheets that teach how to manage emotions helped a bunch.	Uncertain, since all aspects were helpful to me.
Patient 5	All of the tips were very helpful, although I think they will be more helpful once the baby is here than they even were during pregnancy.	I'm not sure if a full 6 weeks of sessions was needed, we seemed to cover a lot in a short amount of time during each session
Patient 6	The wind down activities, set sleeping schedule, and the lesson about sleep/wake regulations	I couldn't edit the Automatic Thought Record file so I had to recreate it. This wasn't a big inconvenience, just the only improvement I can think of.
Patient 7	Cutting out naps, relaxation techniques	None
Patient 8	Expertise, guidance and knowledge from my sleep therapist	I was satisfied with everything
Patient 9	Muscle relaxing excercises Reducing time in bed	Overall the treatment was very satisfactory and it helped me alittle bit to deal with my sleep in a better way and understanding the reasons and possibilities of having insomnia.
Patient 10	tracking my sleep, I never tried that before. When you pay attention to things it is easier to manage.	Not for me personally but I think there are some limitations to the therapy in that if someone is having trouble falling asleep because they are not in a good sleep environment it would be difficult for them to get help. I grew up in Detroit and it was always scary for me to go to sleep at night so I don't know if this approach would have helped me then. I live in the suburbs now and feel very safe so the main issue to tackle was just the fact that I would sleep at random times and it was making me exhausted.
Patient 11	I found each week helpful in a new way. If I had to pick setting a wind down routine, sleep/wake schedule, and what to do if I can't fall sleep or re-fall asleep were most helpful.	I was honestly really happy with all the aspects and suggestions and cognitive therapy.
Patient 12	sleep consolidation and methods to fall back asleep quickly	the cord on the sleep machine could be a little longer.
Patient 13	Breathing techniques and positive ways of thinking were most helpful.	Setting standard bed and awake times. I enjoy awaking and sleeping when i would like.
Patient 14	All of the tips, sleep schedule	None
Patient 15	All of it	None of it

SUPPLEMENT

Table S2. Open-ended, qualitative patient feedback on the helpful and unhelpful components of PUMAS during pregnancy (all quotes remain unaltered).

	PUMAS	
	**What was helpful?**	**What was unhelpful or needs to be improved?**
Patient 1	The meditations	None
Patient 2	Developing a wind down routine. Mediations sent to me via email.	All info provided was useful
Patient 3	The meditations	Nothing
Patient 4	Receiving lots of tools to continue using (primarily the recorded meditations) to help maintain my sleep schedule and health	No suggestions
Patient 5	Meditation and journaling	Sticking to a strict bedtime and wake up time
Patient 6	Having someone who truly understands to talk to	The journaling
Patient 7	Pleasant and unpleasant events worksheet.	Doing the meditations at night while in bed.
Patient 8	Guided meditations to build a foundation over time, experimenting with nighttime routine and bed/wake times; one-on-one sessions each week	Journaling negative events; I think it just wasn't for me. However, the positive events prompts were great!
Patient 9	Sleep restriction, mindfulness, pleasant and unpleasant event journaling	Journaling
Patient 10	30in before bed wind down and nightly awakening meditation to quickly fall back asleep	Journal, I am a very contemplative person and regularly pay attention to the moment and events as they unfold
Patient 11	Guided meditation, sleep schedule	I usually avoid thinking about unpleasant experiences or avoid negative thoughts but week 5 meditation focused on them and made me think more of uncomfortable thoughts. My sleep problem is more related to physical discomfort so other weeks meditations made more sense to me.
Patient 12	setting up a sleep and wake time, not eating when i wake up in the middle of the night, bedtime meditations	I am not sure how the journal entries did or did not affect my sleep
Patient 13	Wind down routine/basic sleep hygiene and learning the concept of sleep pressure to better decide my own bed time and wake time to maximize sleep efficiency. From the beginning, no electronics before bed helped immensely, adding meditations in bed also helped lull me into sleep mode.	I found I was anxious about doing "enough" or being mindful "accurately" I over thought the homework a lot and made myself anxious for nothing. I'm not sure how that could be improved upon, maybe just a general "do your best" statement or an explanation that it's just a means to an end to become more mindful and self evaluate mood/emotions/how we feel in general
Patient 14	Meditation and techniques to fall asleep	None
Patient 15	The tools provided to help my straying thoughts, more refined sleep schedule and peace of mind that i am not alone.	All aspects were great to explore to determine what worked and what didnt.
Patient 16	Meditation, practicing mindfulness, yoga, sleep schedule	wearing sleep tracker doesn't seem relevant
Patient 17	meditations, increasing awareness/ability to be present via pleasant and unpleasant diaries, journal entries	n/a
Patient 18	Wind down period, set bed times/wake times, meditations at bedtime, and understanding the 3 p's and traits I have that make me more susceptible to sleep issues. Also strategies for night awakenings when you feel alert and accepting the present awake period	None
Patient 19	The sticking to the same bed time/wake time has been extremely helpful as well has having guided mediations on hand to us for meditation practice.	Perhaps different types of meditation (with a subtle noise instead of someone speaking) might be helpful for some people. I enjoy white noise/gong sounds and sometimes find a talking voice distracting.
